# Supplementary material for: Therapeutic Efficacy of Arnica in Hamsters with Cutaneous Leishmaniasis Caused by Leishmania braziliensis and L. tropica
Source: Pharmaceuticals (Basel). 2022 Jun 22;15(7):776. doi: 10.3390/ph15070776 (PMC9321785; doi:10.3390/ph15070776)
Supplement: Supplementary file 1 [file pharmaceuticals-15-00776-s001.zip › pharmaceuticals-1751341-supplementary.pdf]

pharmaceuticals

Supplementary Materials

Therapeutic Efficacy of *Arnica* in Hamsters with Cutaneous Leishmaniasis Caused by *Leishmania braziliensis* and *L. tropica*

Sara M. Robledo [1,*], Javier Murillo [1], Natalia Arbeláez [1], Andrés Montoya [1], Victoria Ospina [1], Franziska M. Jürgens [2,†], Iván D. Vélez [1] and Thomas J. Schmidt [2,*,†]

[1] PECET-School of Medicine, University of Antioquia, Calle 70 # 52-21, Medellin 0500100, Colombia; javier.murillo@udea.edu.co (J.M.); natyac182@gmail.com (N.A.); edwin.montoya@udea.edu.co (A.M.); victoriaospina@hotmail.com (V.O.); ivan.velez@udea.edu.co (I.D.V.)

[2] University of Münster, Institute of Pharmaceutical Biology and Phytochemistry, PharmaCampus, Corrensstrasse 48, D-48149 Münster, Germany; franziska.juergens@uni-muenster.de

* Correspondence: sara.robledo@udea.edu.co (S.M.R.); thomschm@uni-muenster.de (T.J.S.); Tel.: +57-304-5762059 (S.M.R.); +49-251-8333378 (T.J.S.)

† Cordially dedicated to Prof. Dr. Andreas Hensel, Münster, Germany, on the occasion of his 60th birthday.

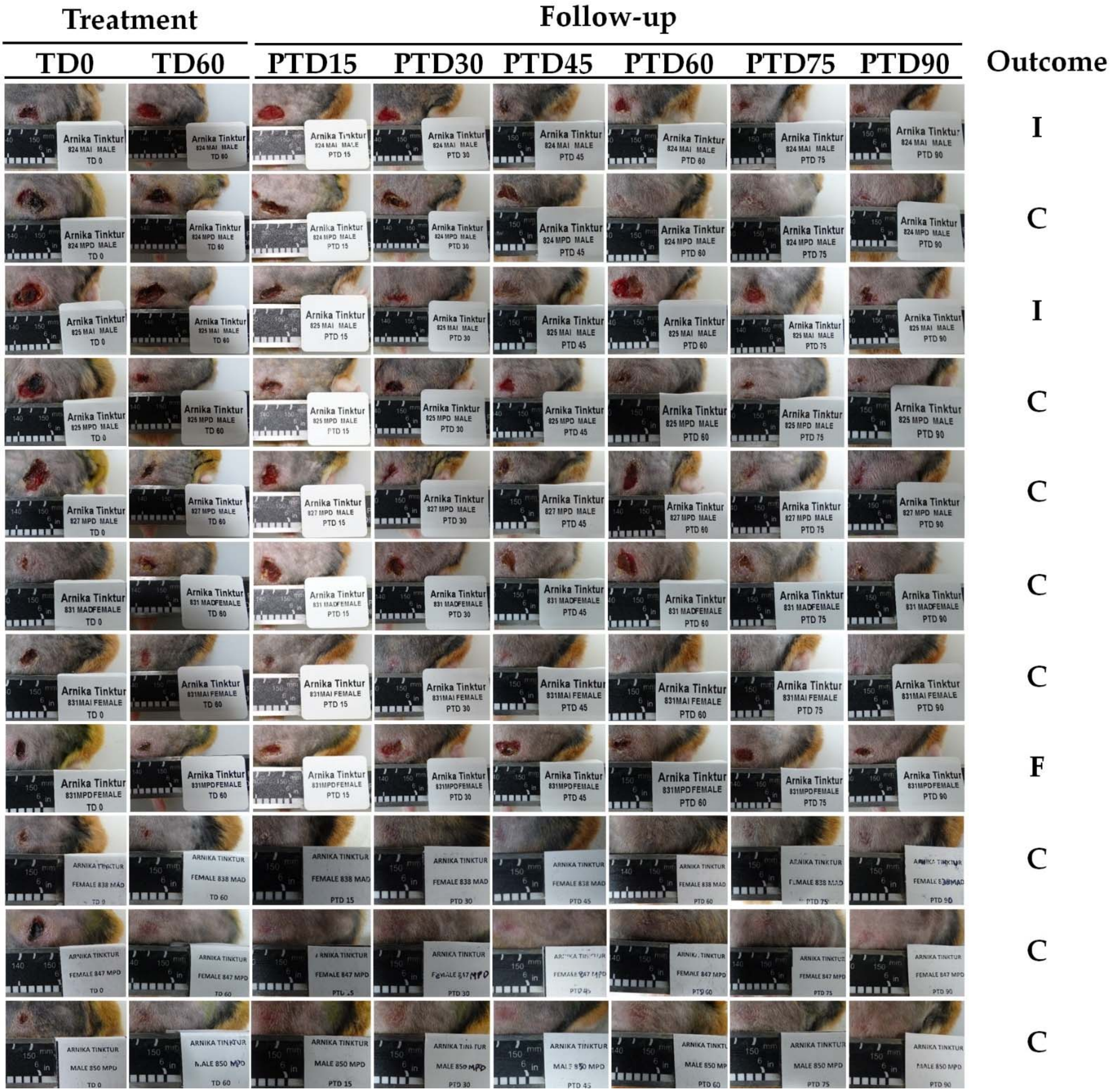

Figure S1. Lesion evolution in hamsters of group **1**. C: cure; I: Improvement; F: Failure.

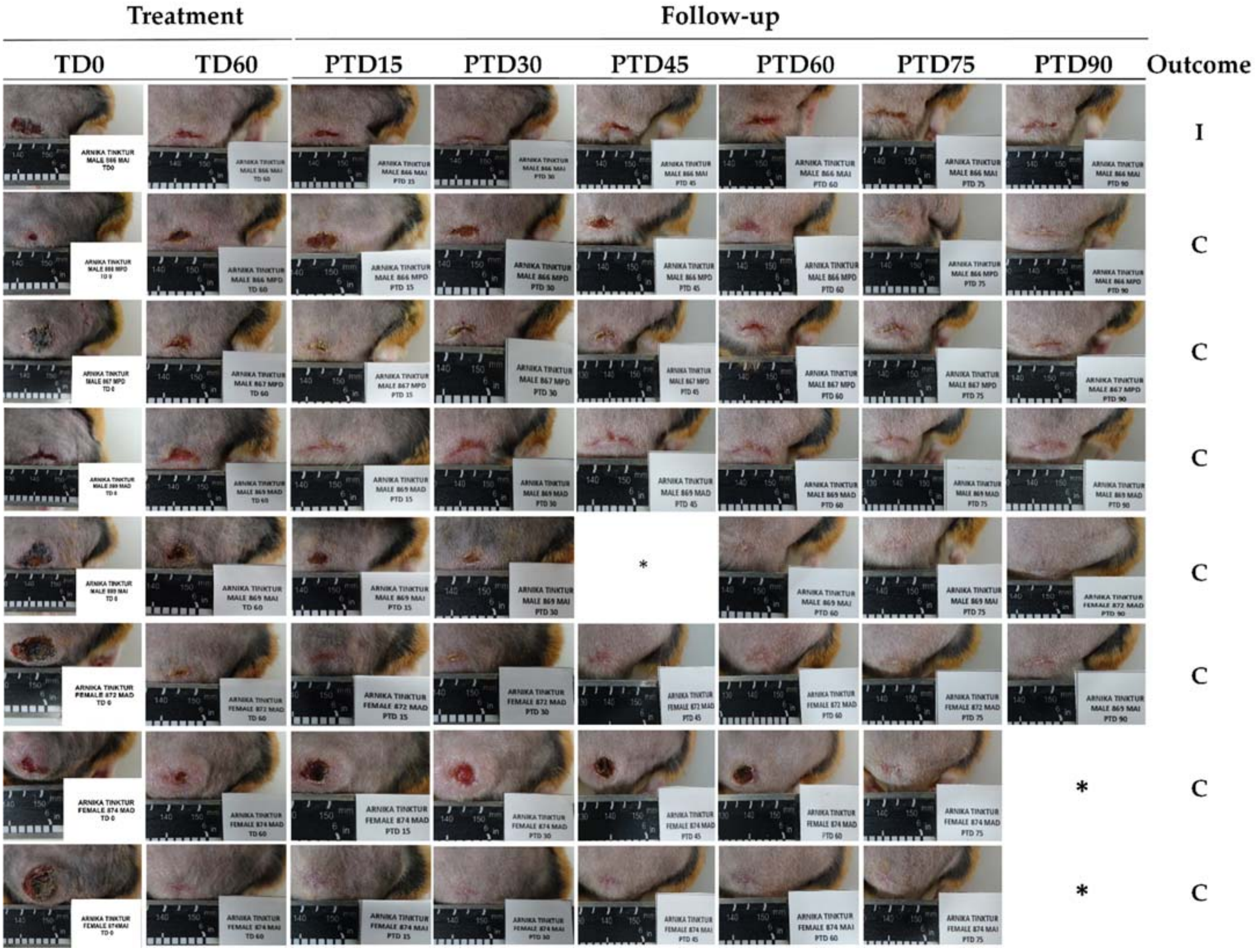

Figure S2. Lesion evolution in hamsters of group **2**. C: cure; I: Improvement; F: Failure; *no photo.

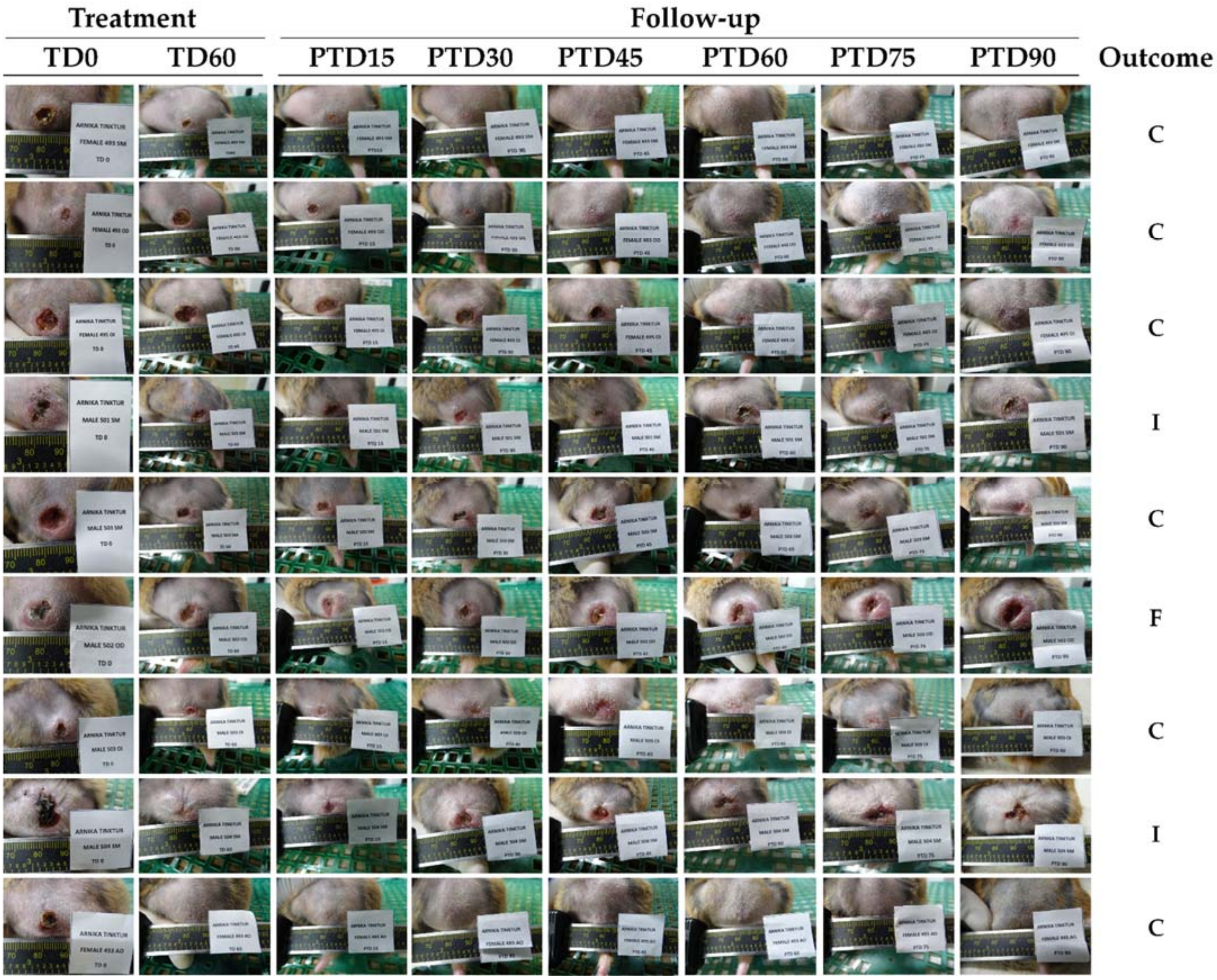

Figure S3. Lesion evolution in hamsters of group **3**. C: cure; I: Improvement; F: Failure.

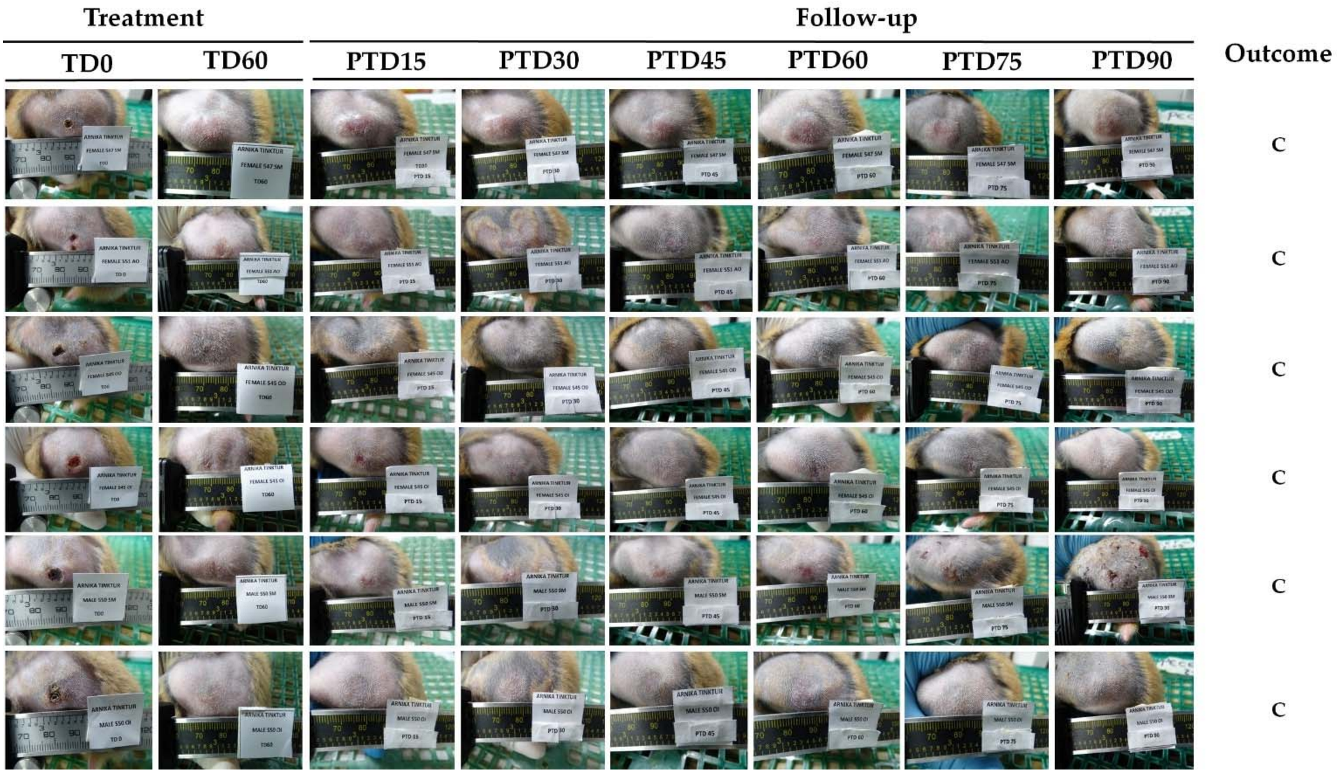

Figure S4. Lesion evolution in hamsters of group **4**. C: cure; I: Improvement; F: Failure.

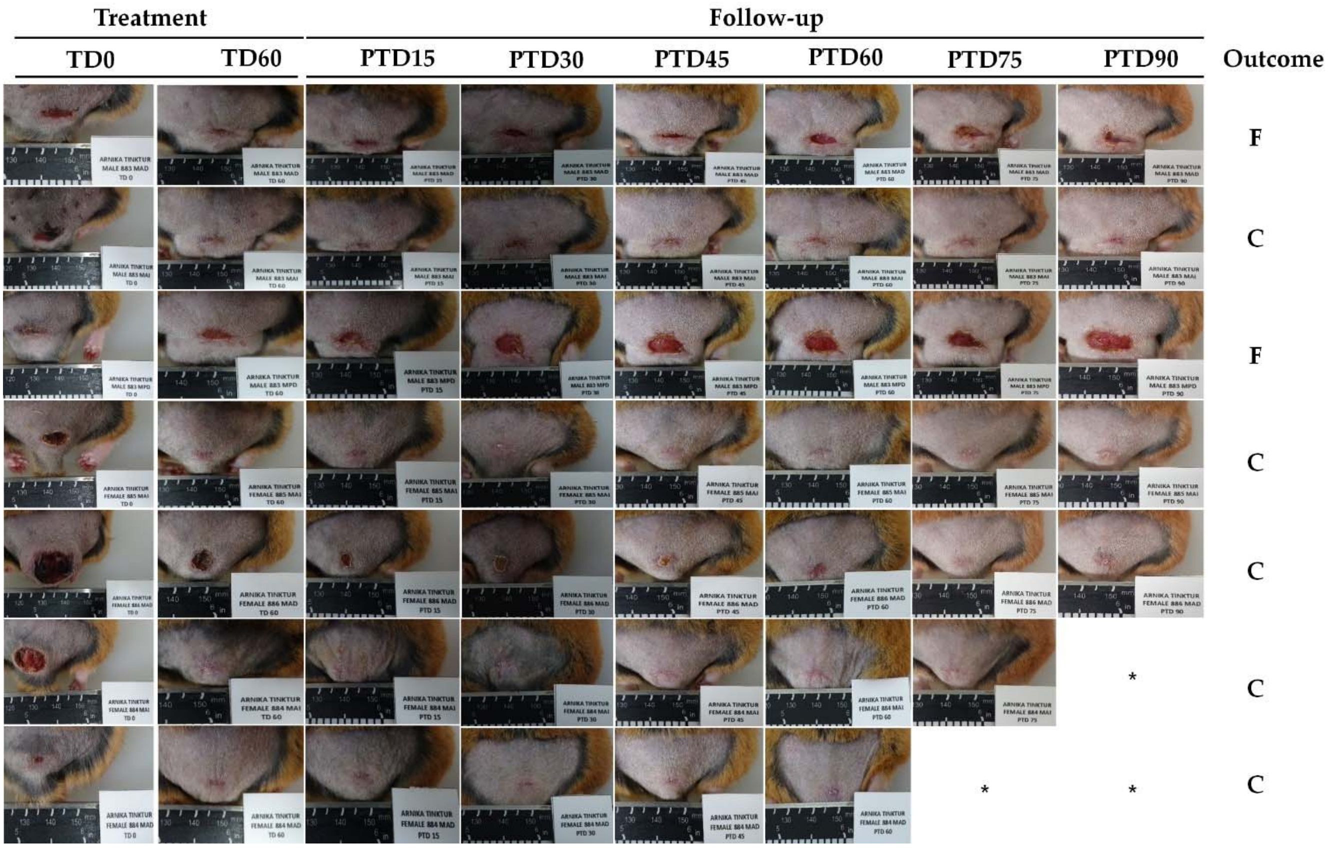

Figure S5. Lesion evolution in hamsters of group **5**. C: cure; I: Improvement; F: Failure; *no photo.

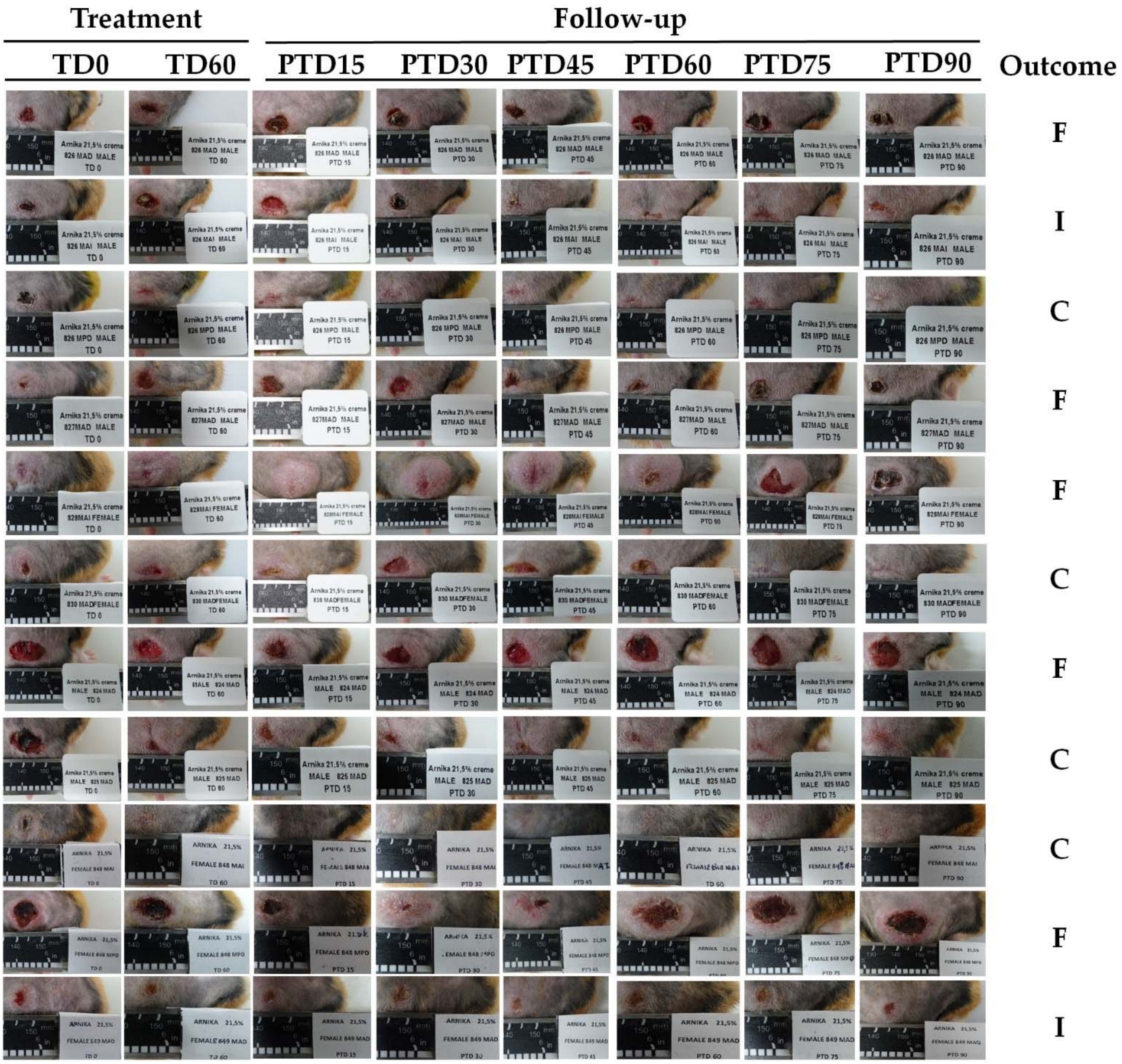

Figure S6. Lesion evolution in hamsters of group **6**. C: cure; I: Improvement; F: Failure.

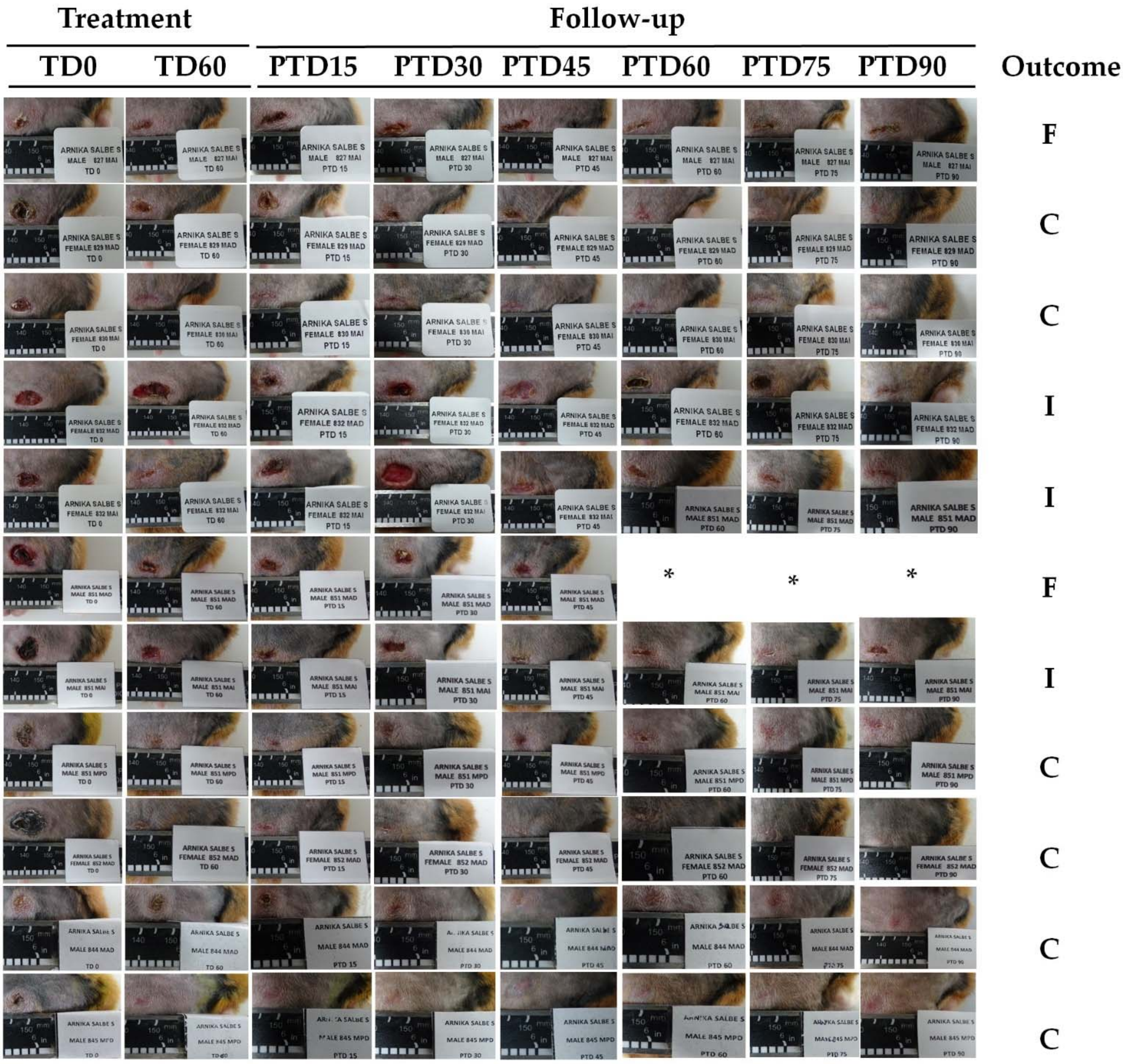

Figure S7. Lesion evolution in hamsters of group **7**. C: cure; I: Improvement; F: Failure; *no photo.

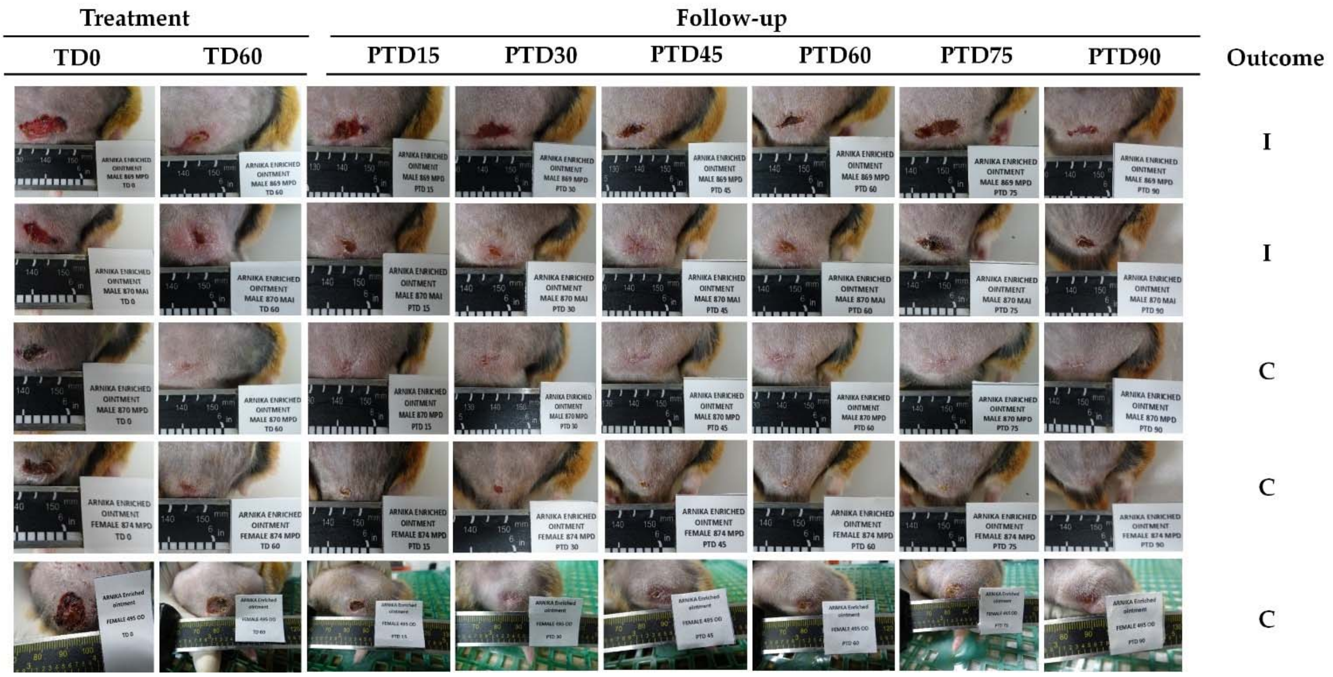

Figure S8. Lesion evolution in hamsters of group **8**. C: cure; I: Improvement; F: Failure.

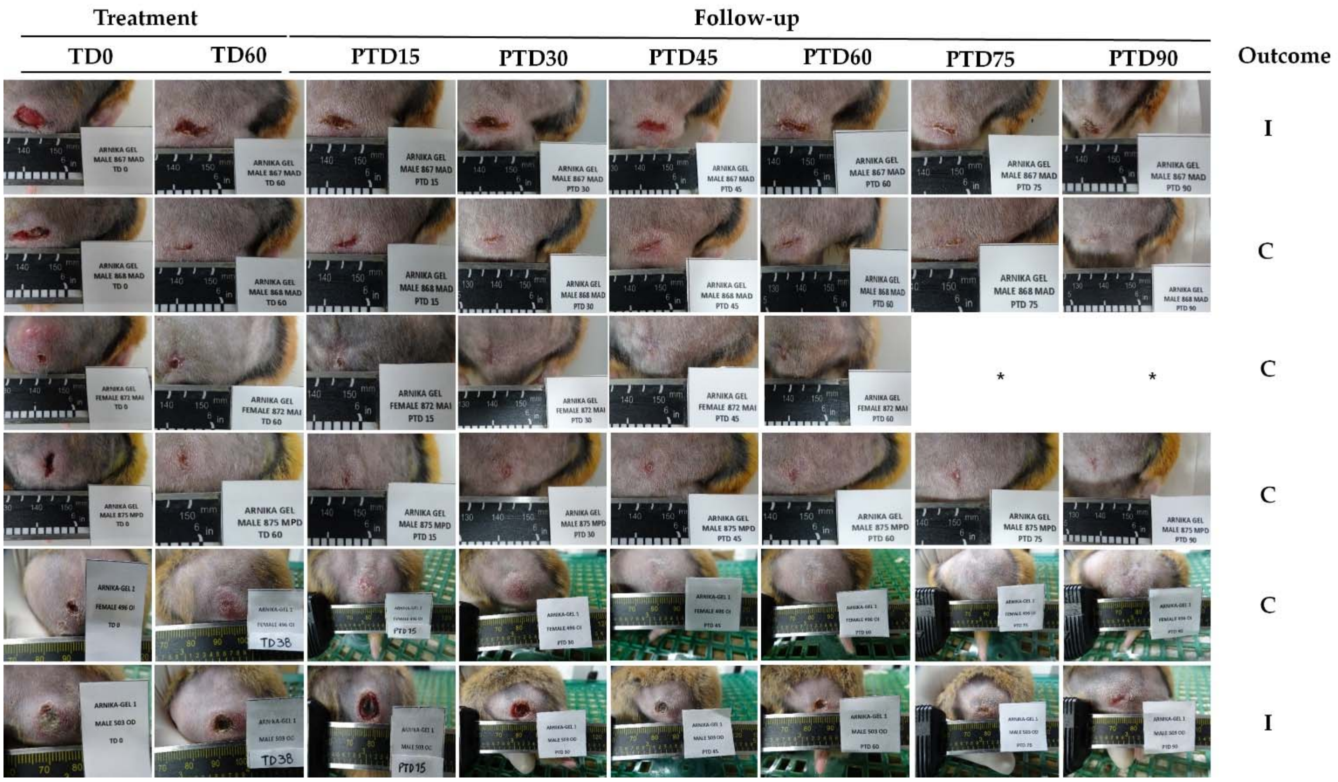

Figure S9. Lesion evolution in hamsters of group **9**. C: cure; I: Improvement; F: Failure; *no photo.

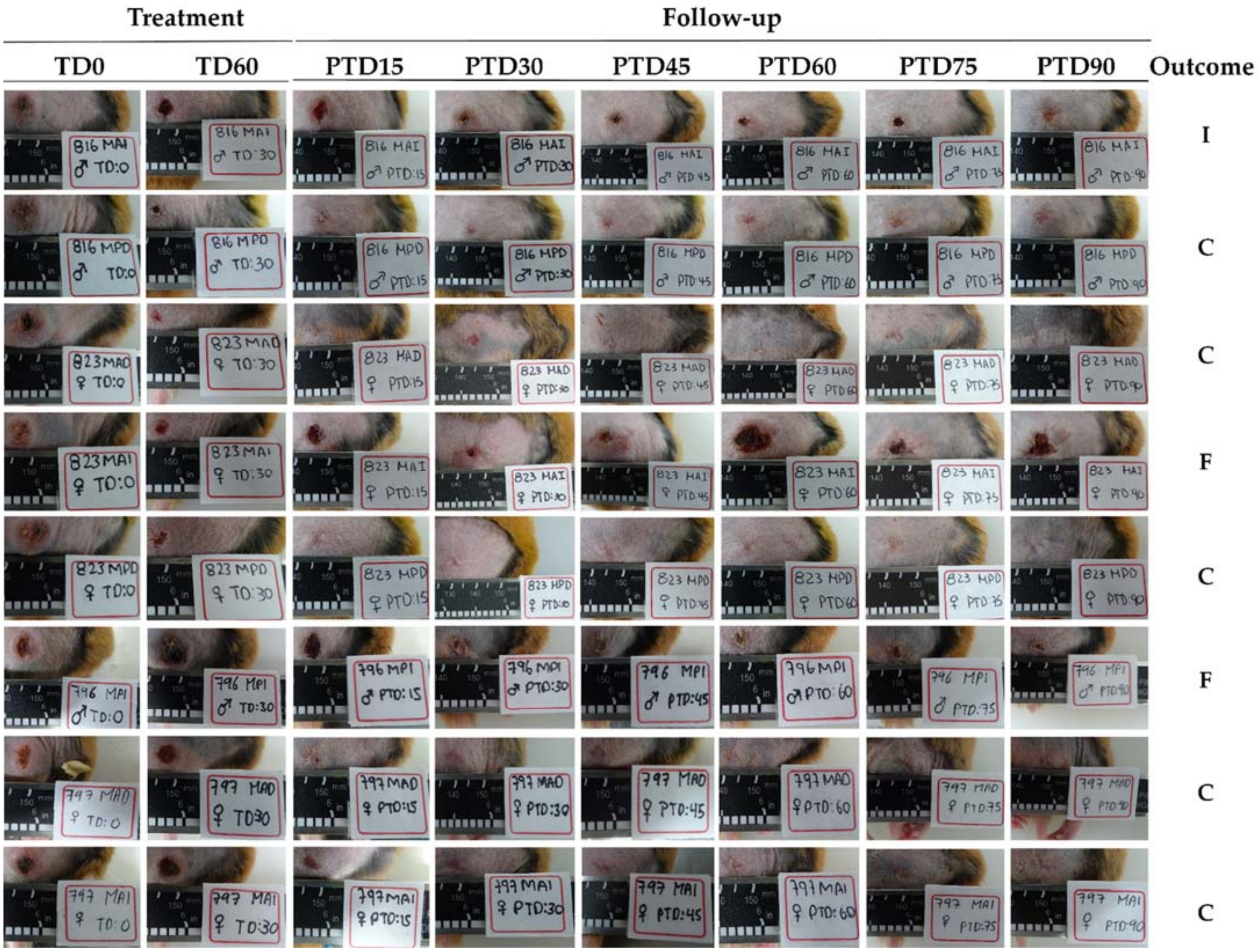

Figure S10. Lesion evolution in hamsters of group **10** (*L. braziliensis*; 200 µg meglumine antimoniate /1x every 3 d, 30 d).
C: cure; I: Improvement; F: Failure

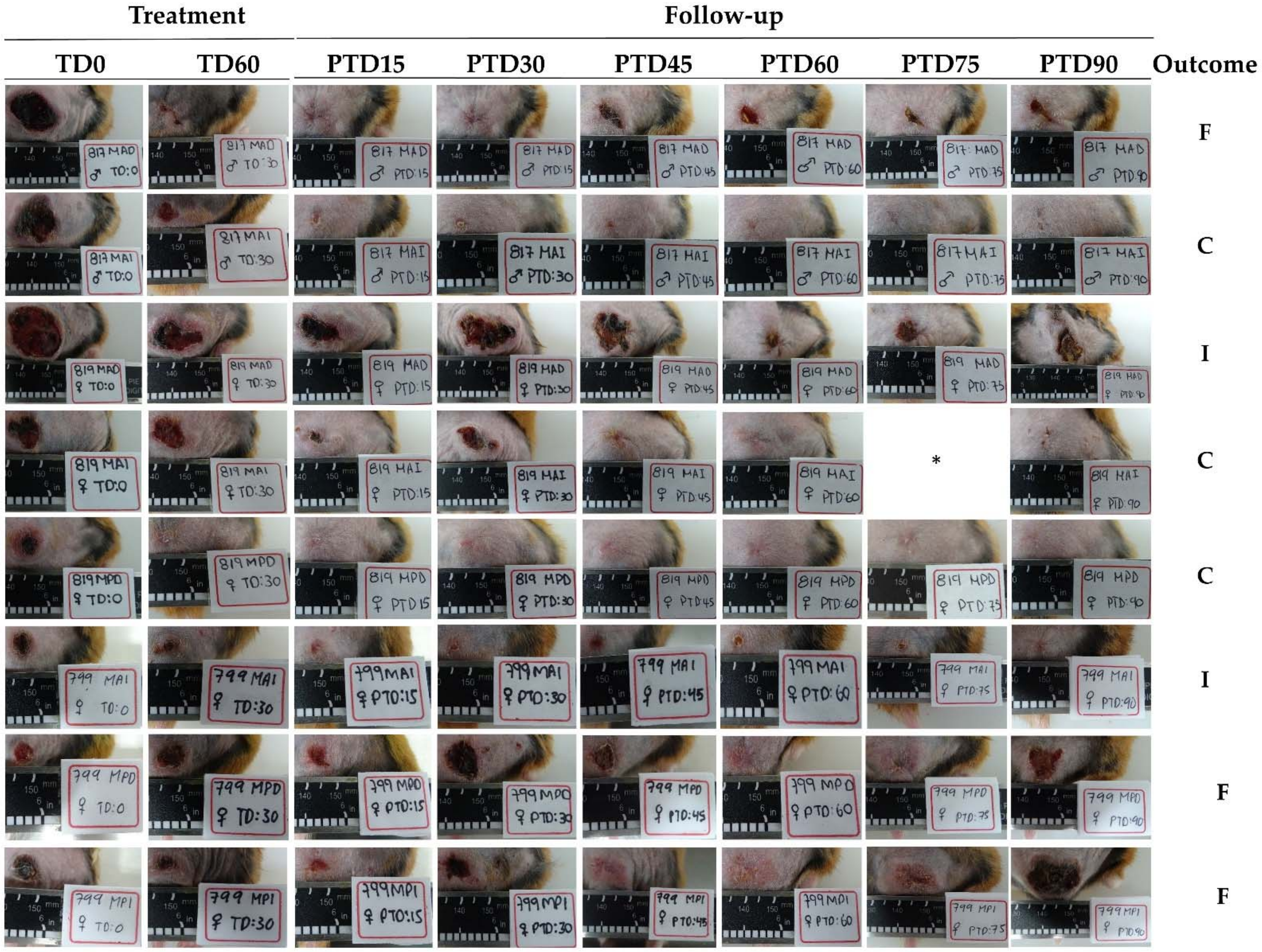

Figure S11. Lesion evolution in hamsters of group **11** (*L. tropica*; 200 µg meglumine antimoniate /1x every 3 d, 30 d).
C: cure; I: Improvement; F: Failure; *no photo.

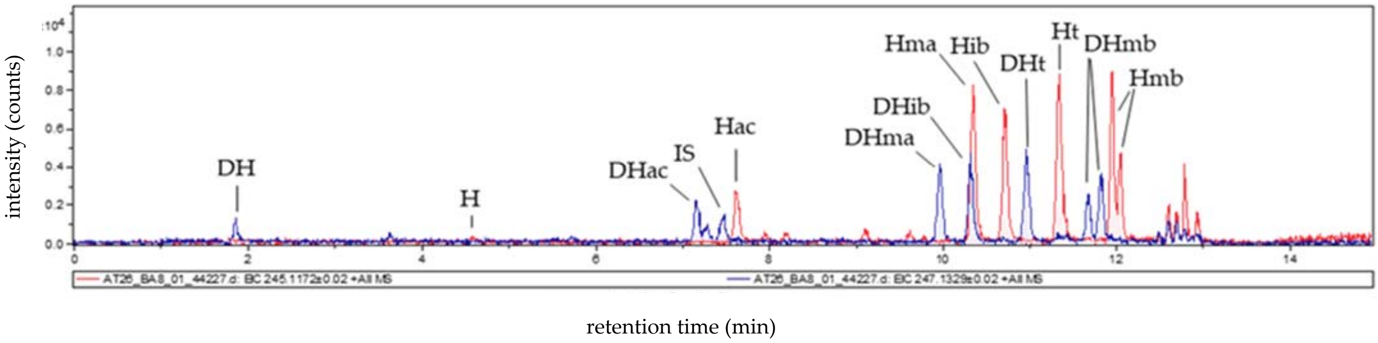

Figure S12: UHPLC-+ESI QqTOF MS analysis of the Arnica tincture under study. Shown are extracted ion chromatograms for the diagnostic molecular skeletal fragments of helenalin derivatives (*m*/*z* 245.1172, red) and 11α,13-dihydrohelenalin derivatives (*m*/*z* 247.1329, blue). Peak labels: DH: 11α,13-dihydrohelenalin; H: helenalin; DHac: 11α,13-dihydrohelenalin acetate; IS: Internal standard α-Santonin; Hac: helenalin acetate; DHma: 11α,13-dihydrohelenalin methacrylate; DHib: 11α,13-dihydrohelenalin isobutyrate; Hma: helenalin methacrylate; Hib: helenalin isobutryrate; DHt: 11α,13-dihydrohelenalin tiglate; Ht: helenalin tiglate; DHmb: 11α,13-dihydrohelenalin-2- and -3-methylbutyrate; Hmb: helenalin-2- and -3-methylbutyrate.

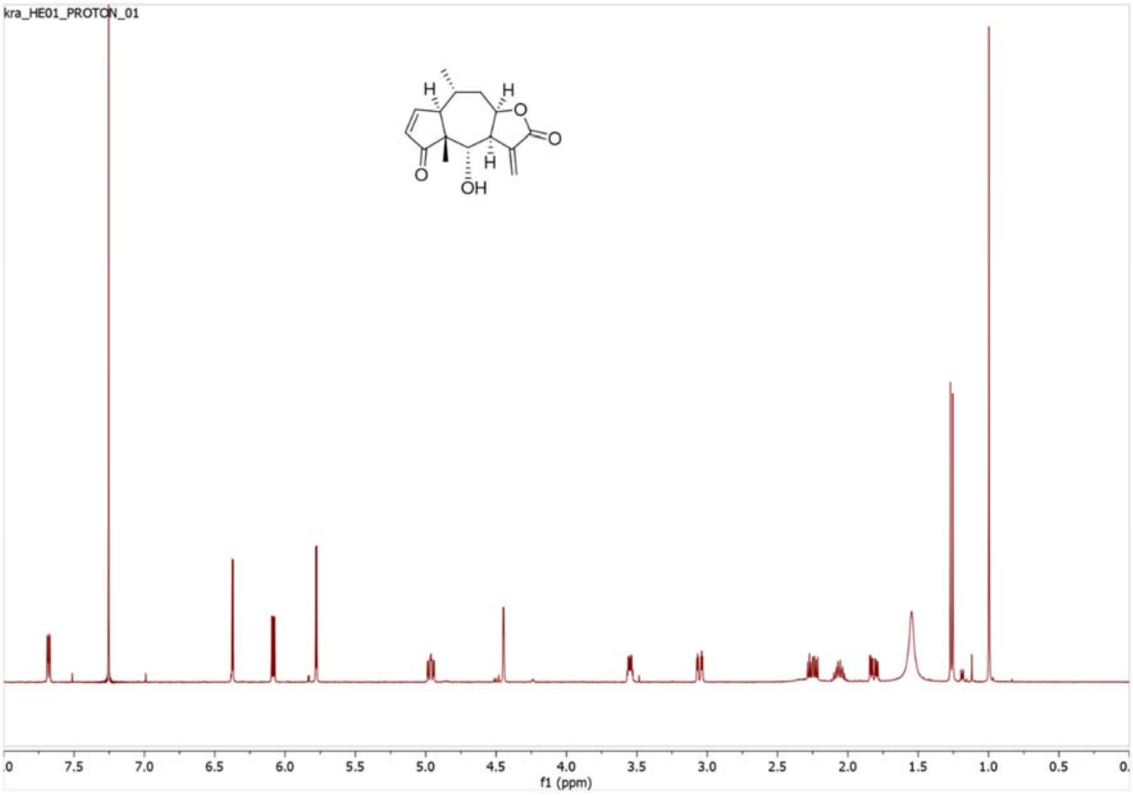

Figure S13: ^{1}H-NMR spectrum of helenalin (600 MHz, $CDCl_3$).

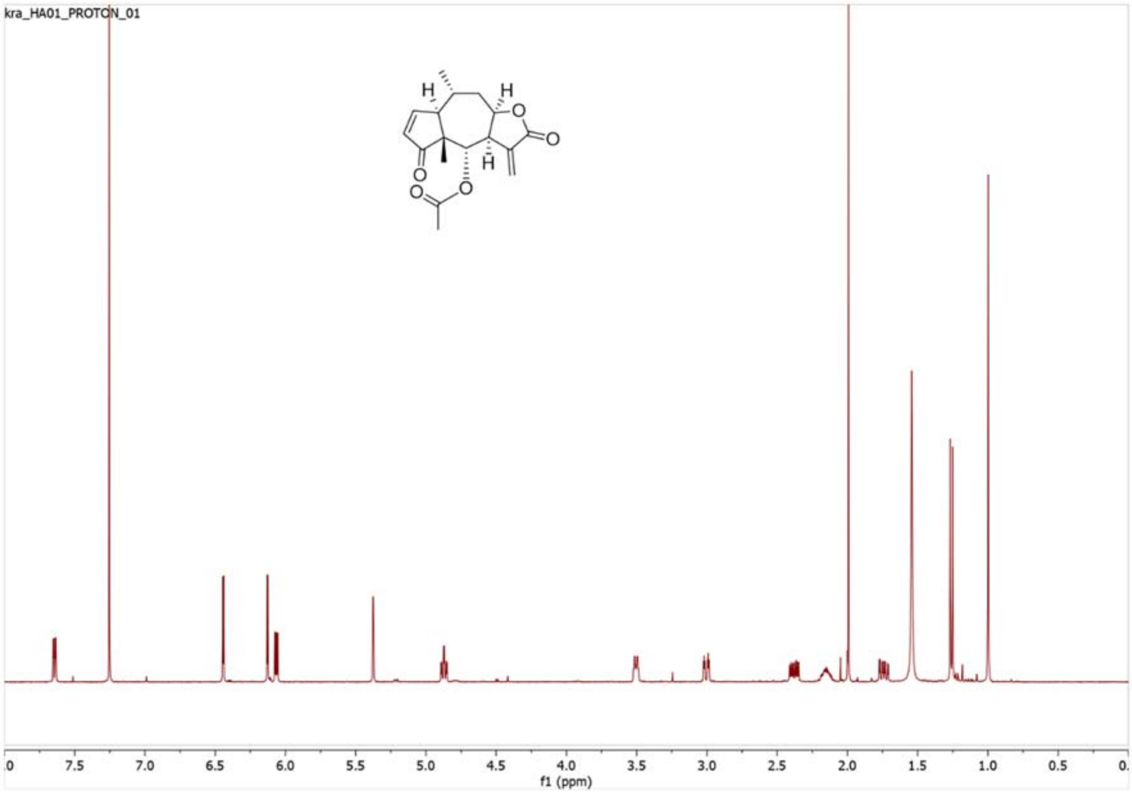

Figure S14: ^{1}H-NMR spectrum of helenalin acetate (600 MHz, $CDCl_3$).

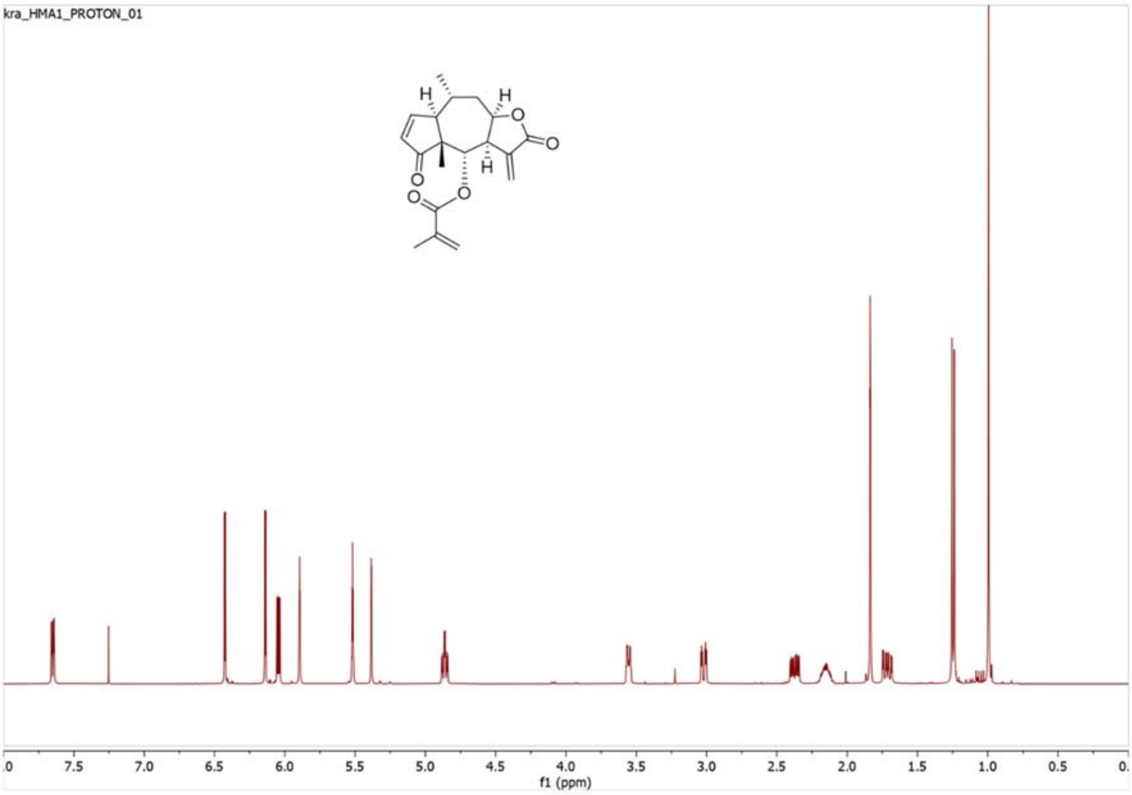

Figure S15: ^{1}H-NMR spectrum of helenalin methacrylate (600 MHz, $CDCl_3$).

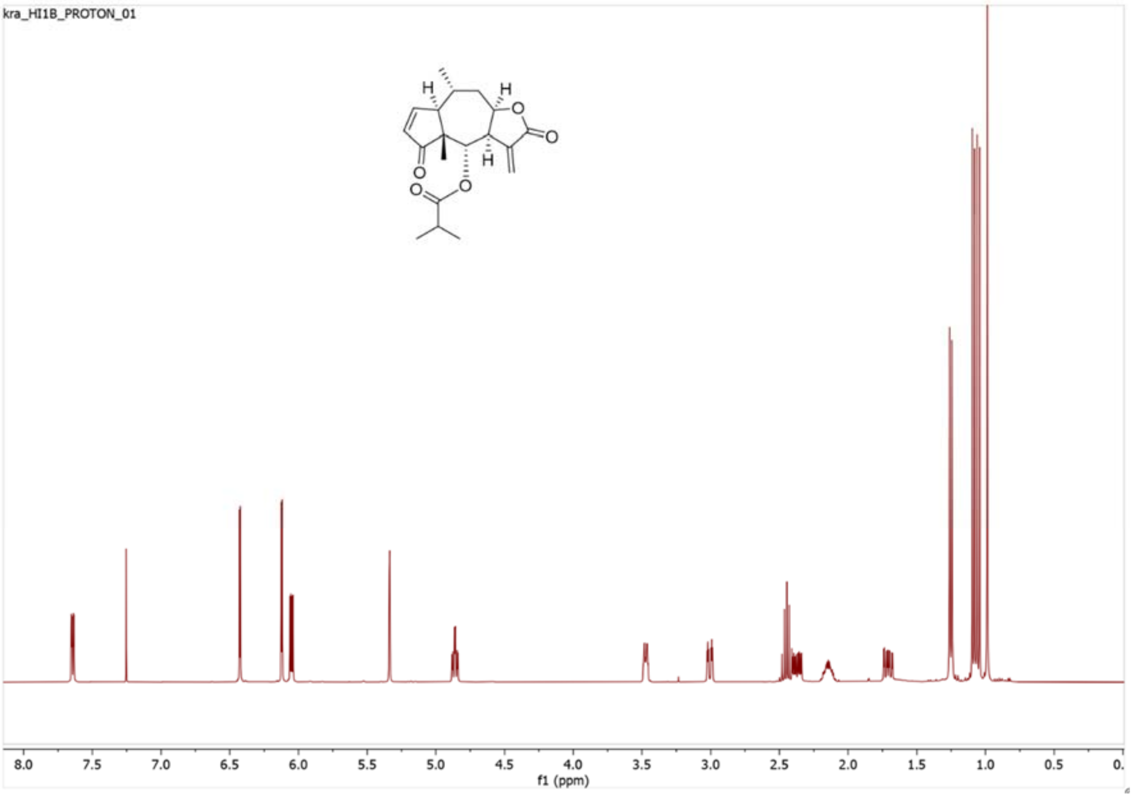

Figure S16: ^{1}H-NMR spectrum of helenalin isobutyrate (600 MHz, $CDCl_3$).

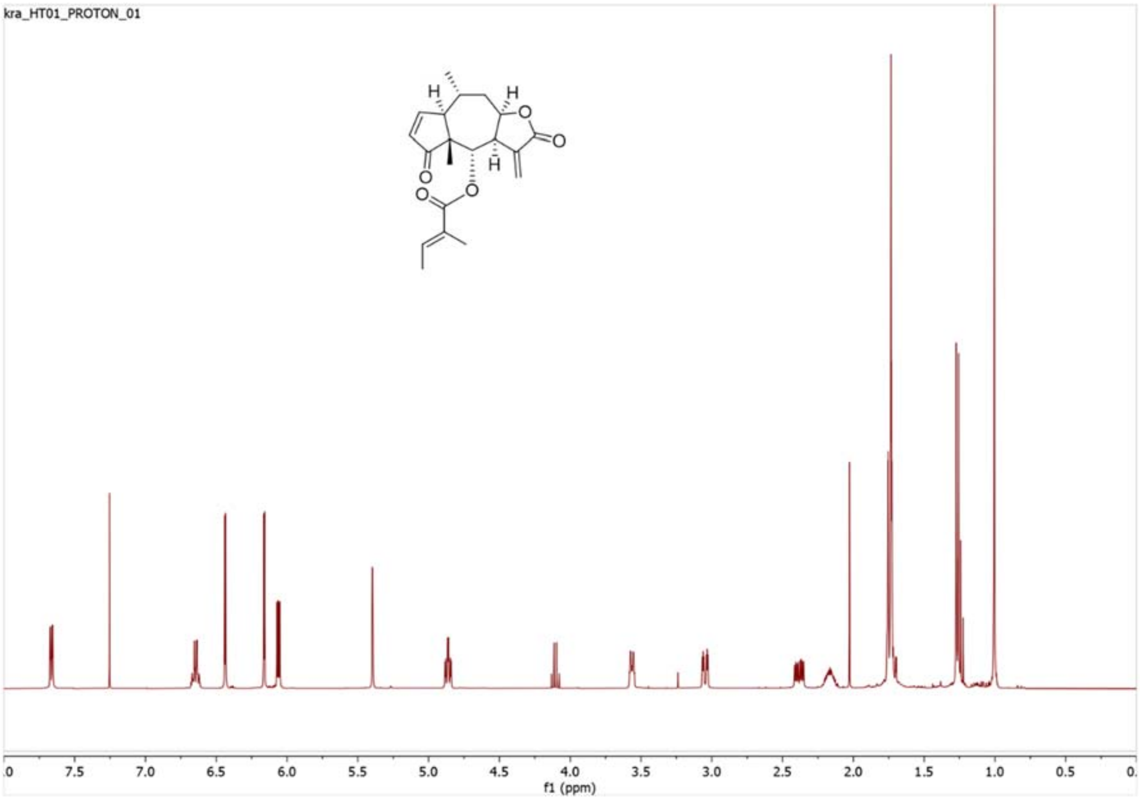

Figure S17: ^{1}H-NMR spectrum of helenalin tiglate (600 MHz, $CDCl_3$).

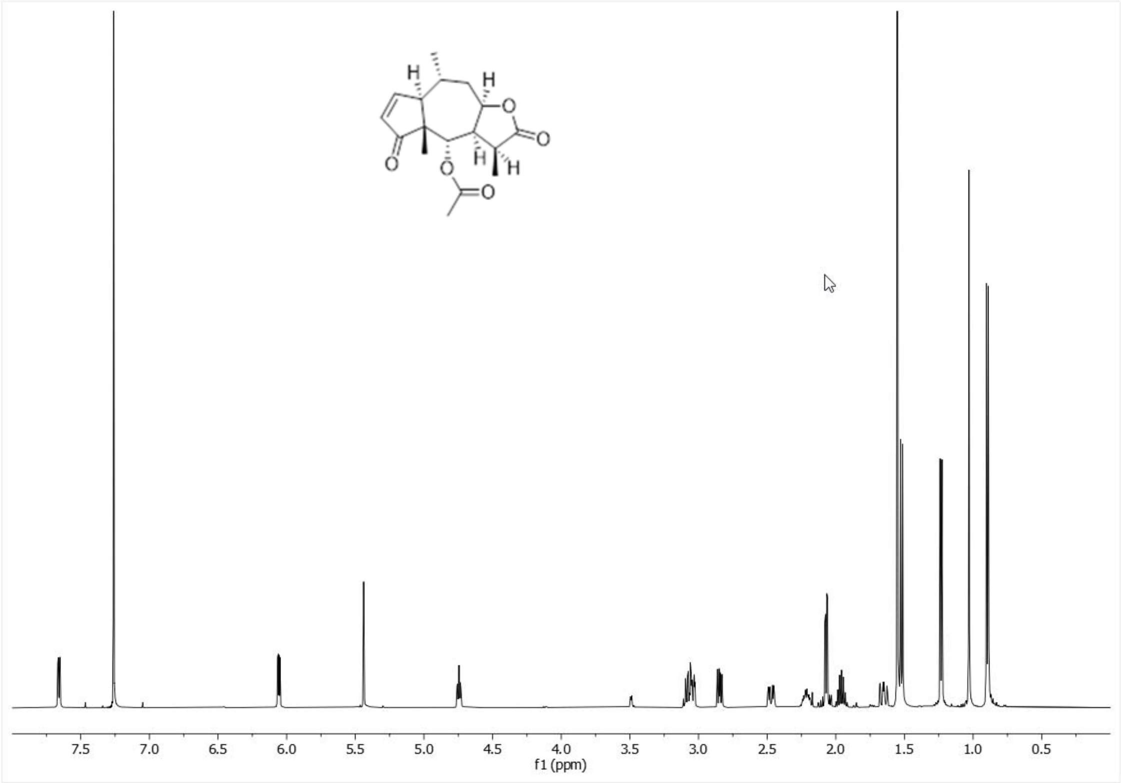

Figure S18: ^{1}H-NMR spectrum of 11α,13-dihydrohelenalin acetate (600 MHz, $CDCl_3$).

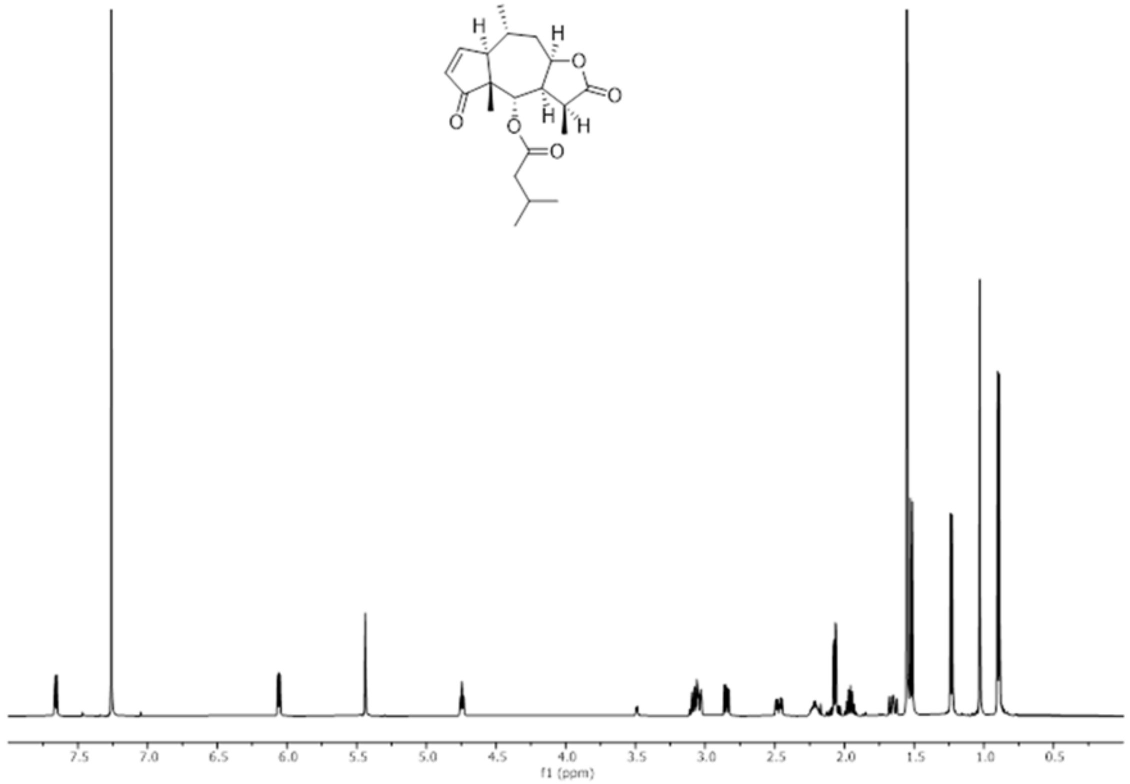

Figure S19: ^{1}H-NMR spectrum of 11α,13-dihydrohelenalin 3-methylbutyrate (=isovalerate; 600 MHz, $CDCl_3$).

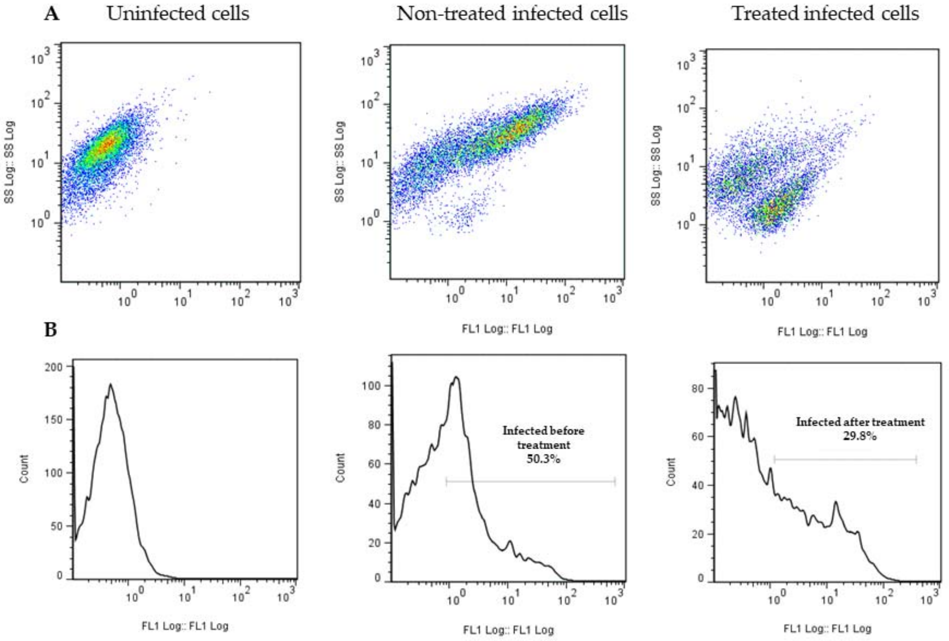

Figure S20: Representative dot-plots (A) and histograms (B) of uninfected, non-treated and treated infected cells.
